# Identification of Aroma Differences in Refined and Whole Grain Extruded Maize Puffs

**DOI:** 10.3390/molecules25092261

**Published:** 2020-05-11

**Authors:** Kenneth Smith, Devin G. Peterson

**Affiliations:** 1 Department of Food Science and Nutrition, 1334 Eckles Avenue, 145 FScN Building, University of Minnesota, St. Paul, MN 55108, USA; smit4423@umn.edu; 2 Department of Food Science and Technology, 2015 Fyffe Rd., 317 Parker Food Science & Technology Building, The Ohio State University, Columbus, OH 43210, USA

**Keywords:** whole grain, refined grain, GC/O, Maillard reaction, maize, aroma, flavor

## Abstract

Differences in the aroma profiles of extruded maize puffs made from refined grain and whole grain flour were investigated. Gas chromatography/mass spectrometry/olfactometry (GC/MS/O) analysis reported 13 aroma compounds with a flavor dilution (FD) value ≥16. Quantitative analysis identified eight compounds as statistically different, of which seven compounds were higher in concentration in the whole grain sample. Sensory recombination and descriptive analysis further supported the analytical data, with higher mean aroma intensities for cooked, corn chip, roasted, and toasted attributes for the whole grain sample. Generally, the compounds responsible for perceived differences in whole grain maize extruded puffs were associated with increased levels of Maillard reaction products, such as 2-ethyl-3,5-dimethylpyrazine and 2-acetyl-2-thiazoline.

## 1. Introduction

The consumption of whole grain has been associated with a range of health benefits such as body-weight regulation, reduced risk of chronic pathological conditions, and reduced blood glucose levels [1,2,3]. However, most Americans fail to consume the recommended whole grain intake (48 g/day), which has a direct effect on health and was recently identified as a main contributor to suboptimal diets that are responsible for 1 out of 5 deaths globally [4,5]. In cereal-based foods, refined grains are often preferred in comparison to their whole grain counterparts. The negative flavor attributes associated with whole grain products including bitter taste and vegetative aromas have been reported as one of the most influential factors limiting consumption [6,7].

Breakfast cereals are cooked products introduced in the human diet at a young age and constitute an excellent opportunity for early exposure to whole grain flavor [8]. Maize is a common grain used for cereal production and the impact of extrusion (cooking) parameters on physico-chemical and sensory properties of extruded cereals has been largely studied [9,10,11,12]. Flavor generation during the extrusion of cereals involves thermally induced reactions, such as the Maillard reaction and lipid degradation. Extrusion conditions such as heat, water content, and residence time have been shown to exert significant effects on the flavor profiles of extruded products [9,12] with cooking temperature identified as a main influential factor for the formation of flavor compounds. Flavor development in extruded products has been investigated with a focus on processing conditions and has overlooked the impact of whole grain versus refine grain flour formulation. In wheat bread, the utilization of whole versus refined grain flour had a significant impact on flavor generation [13]. Several key compounds that give refined wheat bread its typical aroma attributes were less abundant in whole wheat bread due to the suppression of key Maillard-type flavor formation pathways caused by the elevated levels of phenolic compounds. In addition to aroma generation, taste compounds are thermally generated by Maillard-type pathways during bread making [14]. Whole grain flour, as compared to refined grain flour, has elevated levels of phenolic compounds, lipids, vitamins and is composed of a unique protein composition, all of which can significantly alter the thermal generation of flavor compounds [13,15,16,17]. During extrusion processing, high temperature and short time conditions favor Maillard and lipid oxidation flavor-formation pathways [9]; however, the characterization of the flavor differences between whole and refined grain maize products has not been reported.

The objective of this work was to investigate the influence of whole versus refined maize flour on the aroma of extruded puffs and the sensory impact of the identified differences. Aroma-active compounds were identified using gas chromatography/mass spectrometry (GC/MS) and sensory differences were characterized using sensory descriptive analysis.

## 2. Results and Discussion

To characterize the main differences in the aroma profiles of extruded maize puffs made from whole grain versus refined grain flour, odorants were identified using gas chromatography/mass spectrometry/olfactometry (GC/MS/O) and selected based on cut-off flavor dilution (FD) values ≥16 (see Table 1). Thirteen compounds were selected based on this criterion and all the odor compounds identified have been previously reported in extruded maize products [9,12]. However, the influence of flour type on their generation and their impact on the aroma profile of extruded maize whole grain puffs (WGP) and refined grain puffs (RGP) has not been previously reported. Further quantitative analysis of the 13 compounds was conducted and is reported in Table 2.

Eight compounds including six Maillard reaction products and two phenolic compounds were found to be statistically different between WGP and RGP. All the compounds were found in greater amounts in the WGP samples except for 2,5-dimethylpyrazine, which was found in higher concentration in the RGP with 140 µg/kg compared to 100 µg/kg in the WGP. A higher formation of Maillard reaction aroma compounds, in general, in the WGP can be explained by compositional differences between whole and refined grain flours. Milling cereals alters the concentration and composition of proteins and lipids in the flour. The milling process used to produce refined maize flour removes the protein-rich pericarp/germ leaving primarily the starchy endosperm flour [16]. Protein and amino acid content in whole grain maize flour is altered compared to refined maize flour [16,17]. Amino acids are very influential for the progression of Maillard reaction pathways [21,22] and key precursors for the formation of key odorants such as 2-acetyl-2-thiazoline (cysteine [23]) and 2-ethyl-3,5-dimethylpyrazine (alanine [24]). These two aroma compounds showed concentrations 2.2 and 2.1-fold higher in WGP when compared to RGP, respectively. A lower concentration of amino acids (i.e. cysteine and alanine) in refined maize flour could have resulted in the observed changes in the aroma generation noted or perhaps are due to differences in sugar fragmentation. The ratio of precursors, i.e., reducing sugar to *N*-containing compounds, has been demonstrated to selectively favor formation pathways through the modification of the intermediate reactive chemistry [25,26]. In glucose model mixtures in particular, changes in the glucose to amino acid ratio ultimately modulate the generation of pyrazines; a greater ratio of sugars:amino acids in the RGP could have favored the generation of reactive intermediate species involved in the formation of 2,5-dimethylpyrazine, while suppressing other products, such as 2-acetyl-2-thiazoline or 2-ethyl-3,5-dimethylpyrazine.

Two ferulic acid degradation products 2-methoxy-4-vinylphenol and 4-hydroxy-3-methoxybenzaldehyde were quantified in higher amounts in WGP (Table 2) and described in GC/O with clove and vanilla aroma descriptors (Table 1), respectively. In grains, the phenolic material is mainly distributed in the bran layer. For example, in sweet maize the pericarp and germ contain approximately 325 and 702 ug/g of ferulic acid, respectively while the endosperm, the primary component of refined maize flour, contains approximately 13 mg/g [27]. In general, the total phenolic content of the pericarp is approximately 30-fold higher than the endosperm [28]. In bread, the liberation of phenolic compounds (i.e., ferulic acid) from whole wheat flour during baking was reported to suppress the generation of Maillard aroma compounds through carbonyl trapping mechanisms [13]. The noted increase in Maillard aroma compound generation in the WGP versus RGP suggested the phenolic-carbonyl reaction mechanisms that suppressed aroma formation in bread were not relevant in extruded maize products, albeit the heating profile of extrusion cooking is drastically different than baking bread. Others have shown that the addition of the phenolic compound, rutin, during the preparation of baked rye-buckwheat biscuits resulted in higher levels of Maillard-type aroma compounds, such as alkyl-pyrazines [29].

Two lipid oxidation compounds, hexanal and 2-pentylfuran were not found to be statistically different in concentration between RGP and WGP, however (*E*,*E*)-2,4-decadienal was significantly higher (approximately 20%) in the WGP sample. Thus, the higher content of lipid in the whole grain maize did not have a major impact on the formation of these lipid oxidation aroma compounds, perhaps because of the elevated levels of antioxidative components of the pericarp.

**Figure 1:**
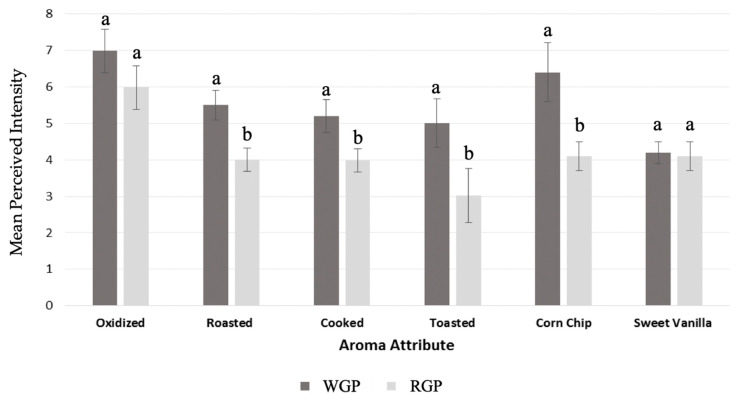
Mean aroma attribute intensity scores of extruded maize refined grain puffs (RGP) and whole grain puffs (WGP); Different letters (a, b) indicate a significant difference between samples for each attribute according to Tukey’s HSD, *p* < 0.05

**Figure 2:**
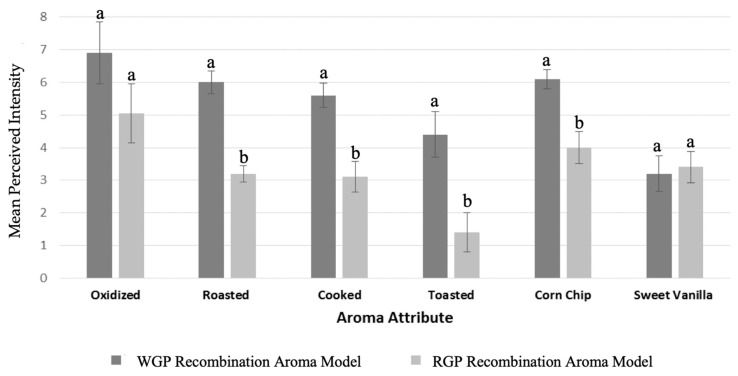
Mean aroma attribute intensity scores of aroma recombination models for extruded maize refined grain puffs (RGP) and whole grain puffs (WGP); Different letters (a, b) indicate a significant difference between samples for each attribute according to Tukey′s HSD, *p* < 0.05.

To draw further insight regarding the impact of quantitative differences of aroma compounds of the maize extruded puffs (Table 2) on the aroma profile, sensory descriptive analysis (DA) was conducted on both the WGP and RGP samples (Figure 1), as well as aroma recombination models (Figure 2).

The perceived intensities of the aroma descriptors were significantly higher in whole grain versus refined maize puffs for four out of six attributes (Figure 1). In the whole grain sample, the highest reported mean intensities were for the attributes cooked, corn chip, roasted, and toasted and likely were associated with the increased concentration of the Maillard-derived compounds (Table 2). All the Maillard compounds, with the exception of 2,5-dimethylpyrazine, were statistically higher in concentration in the WGP compared to the RGP. The oxidized attribute was not rated as significantly different in intensity between WGP and RGP. The lipid oxidation compounds typically associated with oxidized sensory properties including 2-pentylfuran and hexanal were indeed found at similar levels in both samples, whereas 2,4-decadienal was approximately 20% higher in the WGP (Table 2). These results indicated that lipid oxidation did not play a major role in aroma differences between WGP and RGP. Finally, the intensity of the vanilla attribute was rated similarly in both samples, and thus was not established as a discriminant sensory trait common to both WGP and RGP despite higher levels of these phenolic degradation compounds in the WGP sample.

Aroma recombination models were developed to determine the contribution of the aroma composition (Table 2) on the sensory attributes of the WGP and RGP samples (Figure 1). In general, the recombination models of the maize puff samples (Figure 2) agreed with the authentic samples (Figure 1) and showed that toasted, roasted, corn chip, and cooked attributes had significantly higher mean intensities in WGP in comparison to RGP. The odor threshold values are also shown in Table 2, with 10 of the 13 compounds being reported at concentrations above the threshold. Odor thresholds provide a basis to understand sensory relevance; however, some caution would be warranted when extrapolating these threshold values in water to the aroma attributes perceived by the orthonasal evaluation of a low-moisture high-starch puffed cereal product. Nonetheless, when focused on compounds above their aqueous odor threshold values that were also significantly different in concentration (Table 2) and considering the odor properties (Table 1), two Maillard reaction products, 2-ethyl-3,5-dimethylpyrazine and 2-acetyl-2-thiazoline were indicated as the main contributors to the noted sensory differences in the aroma profile of the WGP and RGP samples (Figure 1).

In summary, the Maillard reaction products were established as the main aroma differences between WGP and RGP. This study showed a predominance of Maillard aroma compounds and sensory traits in the WGP likely induced by the heat conditions of the extrusion process. Historically, the aroma attributes of Maillard compounds associated with roasted, toasted, corn chip, and cooked are viewed as positive traits in heat-processed foods. Thus, the aroma attributes of extruded whole grain maize did not appear to negatively alter the flavor profile (compared to refined grain product). However, further work is needed to understand if these changes could contribute to an unbalanced aroma profile when present at higher intensities. Moreover, the impact of whole grain maize on the taste profile (i.e., bitterness) could also play a role in product acceptance.

## 3. Materials and Methods

### 3.1. Materials

Hexanal, 2-methylpyrazine, 2,3-dimethylpyrazine, 2,5-dimethylpyrazine, 2-methyl-2-thiazoline, 2-pentylfuran, 2-ethyl-3,5-dimethylpyrazine, 3-hydroxy-2-methyl-4*H*-pyran-4-one, 2-methylphenol, 2-acetyl-2-thiazoline, (*E,E*)-2,4-decadienal, 2-methoxy-4-vinylphenol, 4-hydroxy-3-methoxybenzaldehyde, 4-heptanone, 2-methyl-3-heptanone, trisodium phosphate, calcium carbonate, anhydrous sodium sulfate, corn starch and sodium chloride were purchased from Sigma-Aldrich (St. Louis, MO, USA). Methanol and methylene chloride, in GC-Resolv^®^ grade, were purchased from Fischer Scientific (Pittsburgh, PA, USA). Maizewise™ whole grain and Innovasure™ refined maize flours were purchased from Cargill (Minneapolis, MN, USA). All the sensory reference materials, dry uncooked Bob′s Red Mill Steel Cut Oats, Bergen Unsalted Lightly Roasted Almonds, Organic Valley Whole UHT Milk, Toasted Wonder Bread™, Nilla^®^ Wafers, and Old Dutch Restaurante^®^ Style Yellow Corn Tortillas Chips, were purchased from a local grocery store.

### 3.2. Twin-Screw Extrusion

Extrusion conditions were designed to yield uniform cell structure throughout each puff [10]. Briefly, extrusion processing was carried out using The Joseph J. Wartheson pilot plant (Department of Food Science and Nutrition, University of Minnesota, St. Paul, MN) with a Buhler DNDL-44 twin-screw extruder (Uzwil, Switerland). Two formulations were produced, a refined maize flour formulation and a whole grain maize flour formulation. The refined maize flour dry formulation consisted of 970 g (97%) refined maize flour with 10 g (1%) trisodium phosphate, 10 g (1%) calcium carbonate, and 10 g (1%) sodium chloride. The whole grain maize formulation consisted of 465 g (48%) refined maize flour and 505 g (52%) whole grain maize flour with 10 g (1%) trisodium phosphate (TSP), 10 g (1%) calcium carbonate, and 10 g (1%) sodium chloride. The ingredients were added to a mixer and mixed for 10 min. The mixture was added with 14% (*w*/*w*) water into the extruder with a low work screw configuration via a feeder and processed per the following extrusion parameters: computer-controlled shaft speed of 350 rpm, measured die pressure of 10.1 ± 0.5 bar, die temperature of 160 ± 1 °C, material throughput of 50.8 ± 0.1 kg/h with 7 kg/h water, and a cutter speed of 1200 rpm. Due to differences in the physical and chemical characteristics of the refined and the whole grain flour mixes, the refined maize flour formulation showed an increased shaft torque of 224 N·m over the whole grain maize flour formulation, which had a shaft torque of 215 N·m. The specific mechanical energy for refined maize flour formulation was 164 kW/h, while the whole grain maize flour was 159 kW/h. Other parameters were constant across both formulations. The puffed product was collected, dried on a liquid air bed, and stored in high-density polyethylene bags at –40 °C for later analysis.

### 3.3. Solvent Extraction

Briefly, 300 g of maize puffs were ground and placed in a 1 L Erlenmeyer flask. Next, 600 g of methanol spiked with 0.1 mg/L 4-heptanone were added to the flask, which was then shaken for 24 h on an orbital shake table set at 200 rpm. Methanol was collected and the ground maize puffs were re-extracted for 2 h using 400 g of methanol at 200 rpm. Organic layers were pooled, and 600 g of the methanol collected was subsequently combined with 600 mL of reverse osmosis purified water. The water-methanol mixture was then poured into three 1 L separatory funnels and extracted using 500 g of methylene chloride (DCM) spiked with 0.1 mg/L of 2-methyl-3-heptanone. DCM was added in 100 mL aliquots to each funnel for a total of 5 extractions. The DCM extract was then placed in a –20 °C freezer overnight to separate and remove any residual water-methanol. The DCM extract was collected and then dried using sodium sulfate and subsequently concentrated via distillation to 1.0 g. The concentrated extract was stored at –80 °C until analysis. Additionally, internal standards used were analyzed for reproducibility during extraction. Methanol was spiked with 4-heptanone and DCM was spiked with 2-methyl-3-heptanone to achieve 100 and 150 mg/L, respectively, in the concentrated solvent. This protocol, when compared to DCM extraction, resulted in 30% more aroma actives detected during GC/O (data not shown).

### 3.4. Gas Chromatography/Olfactometry/Mass Spectrometry (GC/O/MS): Aroma Extraction Dilution Analysis (AEDA)

GC/O analyses were performed on an HP6890 GC (Agilent Technologies, Santa Clara, CA, USA) equipped with a DB-5 column (30 m × 0.25 mm i.d. × 0.25 µm film thickness (Agilent Technologies)) coupled with a 5973 MS (Agilent Technologies) operated in electron impact mode as similarly described by Moskowitz et al. [13]. The system was also equipped with an olfactometry port (Gerstel, Mülheim an der Ruhr, Germany). The effluent was divided 1:1 between the MS and the olfactometry port. The GC conditions were as follows: 0.5 µL sample was injected via air sandwich technique into the inlet which was held at 250 °C set to splitless mode, helium carrier gas was at a constant pressure of 180 kPa. The GC oven temperature program was as follows: initial conditions 40 °C held for 2 min, followed by a 7 °C/min ramp until 250 °C, which was held for 10 min. Each sample was diluted by half-volume in dichloromethane until the dilution had been carried out to a concentration of 128th of the original extraction had been achieved. The largest dilution at which each compound was detected was defined as the FD value. Each dilution was analyzed in triplicate by two panelists. Compound identification was performed using mass spectral data, odor descriptors, and the linear retention index (LRI) of the authentic compound. LRI values were calculated using an *n*-alkane ladder.

### 3.5. GC/MS Identification and Quantitation

The GC/MS analysis was performed using a 7890 GC (Agilent Technologies) coupled to a time of flight (TOF) MS (LECO Pegasus 4D, St. Joseph, MI, USA). The isolate was analyzed on two alternate column chemistries, namely DB-5 and DB-Wax. For the DB-5 analysis analogous column and oven conditions were as previously described. For the DB-Wax (60 m × 0.25 mm i.d. × 0.25 µm film thickness, Agilent Technologies) the GC conditions were as follows: 0.5 µL was injected into an inlet heated to 250 °C. The GC oven temperature program was as follows: initial conditions 40 °C followed by a 5 °C/min ramp to 250 °C and then held for 10 min, flow at 1 mL/min.

Quantification was carried out using five-point calibration curves for each of the 18 compounds in the following concentration ranges (µg/kg) listed, hexanal (50–800), 2-methylpyrazine (55–80), 2,3-dimethylpyrazine (51–815), 2,5-dimethylpyrazine (54–860), 2-methyl-2thiazoline (61–975), 2-pentylfuran (43.5–775), 2-ethyl-3,5-dimethylpyrazine (52.5–840), 3-hydroxy-2-methyl-4*H*-pyran-4-one (44–700), 2-methylphenol (60–965), 2-acetyl-2-thiazoline (61–975), (*E*,*E*)-2,4-decadienal (92.5–1480), 2-methoxy-4-vinylphenol (438–7000), 4-hydroxy-3-methoxybenzaldehyde (612–10000), all curves had high linearity (R^2^ > 0.98 for all compounds) as similarly descripted by Trikusuma et al. [30].

### 3.6. Sensory Evaluation

The aroma of the maize puffs was evaluated by 12 trained panelists (4 male and 8 female, ages 22–32) from the University of Minnesota Department of Food Science and Nutrition (St. Paul, MN, USA). Training consisted of 10 sessions of 1 h. The first training session was dedicated to lexicon development and selection of references. Panelists generated the six following descriptive terms: oxidized, roasted, cooked, toasted, vanilla, and corn chip. Representative food samples were selected as references for sensory attributes: dry uncooked Bob′s Red Mill Steel Cut Oats represented the oxidized aroma, Bergen Unsalted Lightly Roasted Almonds represented the roasted aroma, Organic Valley Whole UHT (ultra-heat treated) Milk represented the cooked aroma, toasted Wonder Bread™ represented the toasted aroma, Nilla^®^ Wafers represented the vanilla aroma, and Old Dutch Restaurante^®^ Style Yellow Corn Tortillas Chip represented the corn chip aroma. The recombination samples were prepared by adding 10 μL of the aroma compound mixture (in ethanol) to 15 g corn starch at the levels quantified in refined and whole grain maize puff samples (Table 2) in sealed 50 mL amber glass containers with Teflon^®^ lined lids. The recombination samples were allowed to equilibrate for 12 h and mixed in a drum tumbler prior to evaluation. Panelists were asked to assess the intensity of the six aroma descriptors orthonasally on a 0–15 pt scale with 0 being not noticeable and 15 being intense. All samples and recombination samples were evaluated in duplicate. For each replicate, a new sample bottle was analyzed. Data were analyzed using analysis of variance and Tukey′s HSD test with a probability of *p* ≤ 0.05. The effect of replicate and the panelist–sample interaction was not significant, indicating that data collected were reproducible and that panel was aligned toward the sensory attributes. Data were processed using SPSS Statistics (IBM, Armonk, NY, USA).

## Figures and Tables

**Table 1:** Identified aroma compounds in extruded maize refined grain puffs (RGP) and whole grain puffs (WGP).

Compound ^a^	Odor Descriptor ^b^	LRI ^c^		Flavor Dilution Value ≥ 16 ^d^	
		DB-Wax	DB-5	WGP	RGP
hexanal	Green/Oxidized	1084	801	64	32
2-methylpyrazine	Roasted	1176	827	128	64
2,3-dimethylpyrazine	Roasted	1240	911	128	32
2,5-dimethylpyrazine	Roasted	1253	912	16	32
2-methyl-2-thiazoline	Roasted/Toasted	1436	933	128	64
2-pentylfuran	Earthy/Oxidized	1240	993	128	32
2-ethyl-3,5-dimethylpyrazine	Roasted	1457	1081	64	32
3-hydroxy-2-methyl-4*H*-pyran-4-one	Caramel/Toasted	1955	1087	128	64
2-methoxyphenol	Smokey	1872	1088	128	64
2-acetyl-2-thiazoline	Popcorn/Corn Chip	1772	1103	64	32
(*E*,*E*)-2,4-decadienal	Oxidized	1815	1312	64	32
2-methoxy-4-vinylphenol	Clove	2189	1322	128	64
4-hydroxy-3-methoxybenzaldehyde	Vanilla-like	2589	1410	64	16

^a^ Compounds positively identified by linear retention index (LRI), mass spectrometry (MS), and authentic compound; ^b^ Odor described at sniffing port during gas chromatography/olfactometry (GC/O), ^c^ LRIs calculated using GC-MS on DB-WAX and DB-5 columns, values relatively to the n-alkane ladder, ^d^ Flavor dilution based on the average of two panelists.

**Table 2:** Concentration of aroma compounds in extruded maize refined grain puffs (RGP) and whole grain puffs (WGP).

Compound	Mean ± CV ^2^		Concentration Ratio (WGP/RGP)	Odor Threshold in Water (µg/L) [18,19,20]
	WGP (µg/kg)	RGP (µg/kg)		
hexanal ^1^	470± 8	436 ± 24	1.1	4.5–5.0
2-methylpyrazine	363± 7 ^b^	292± 3 ^a^	1.2	60,000–105,000
2,3-dimethylpyrazine	653± 7 ^b^	280± 2 ^a^	2.3	2,500–35,000
2,5-dimethylpyrazine	100± 8 ^b^	141± 5 ^a^	0.7	800–1800
2-methyl-2-thiazoline ^1^	147± 10	137± 13	1.1	2
2-pentylfuran ^1^	183± 12	153± 9	1.2	6
2-ethyl-3,5-dimethylpyrazine ^1^	260± 16 ^b^	124± 18 ^a^	2.1	1
3-hydroxy-2-methyl-4*H*-pyran-4-one	370± 9 ^b^	321± 10 ^a^	1.2	35,000
2-methoxyphenol ^1^	317± 5	297± 15	1.1	3–21
2-acetyl-2-thiazoline ^1^	377± 5 ^b^	168± 10 ^a^	2.2	1
(*E*,*E*)-2,4-decadienal ^1^	293± 14^b^	243± 3 ^a^	1.2	0.07
2-methoxy-4-vinylphenol ^1^	3600± 4 ^b^	843± 3 ^a^	4.3	3
4-hydroxy-3-methoxybenzaldehyde ^1^	3517± 7 ^b^	1218± 5 ^a^	2.9	20–200

^1^ Concentration above aqueous odor threshold values; ^2^ Different letters (a, b) indicate a significant difference between samples using a t-test, *p* < 0.05, *n* = 5.
